# Preventing Surgical Site Infections Using a Natural, Biodegradable, Antibacterial Coating on Surgical Sutures

**DOI:** 10.3390/molecules22091570

**Published:** 2017-09-19

**Authors:** Jochen Reinbold, Ann-Kristin Uhde, Ingrid Müller, Tobias Weindl, Jürgen Geis-Gerstorfer, Christian Schlensak, Hans-Peter Wendel, Stefanie Krajewski

**Affiliations:** 1 Department of Thoracic, Cardiac and Vascular Surgery, University Hospital Tuebingen, 72076 Tuebingen, Germany; stefanie.krajewski@uni-tuebingen.de (J.R.); ann-kristin.uhde@student.uni-tuebingen.de (A.-K.U.); christian.schlensak@med.uni-tuebingen.de (C.S.); hans-peter.wendel@med.uni-tuebingen.de (H.-P.W.); 2 Department of Pharmaceutical Engineering, Albstadt-Sigmaringen University, 72488 Sigmaringen, Germany; mueller@hs-albsig.de; 3 Aimecs® GmbH, 84347 Pfarrkirchen, Germany; tobias.weindl@aimecs.de; 4 Section Medical Materials Science & Technology, University Hospital Tuebingen, 72076 Tuebingen, Germany; juergen.geis-gerstorfer@uni-tuebingen.de

**Keywords:** surgical site infection, antibacterial coating, suture, biodegradable, totarol, *Staphylococcus aureus*

## Abstract

Surgical site infections (SSIs) are one of the most common nosocomial infections, which can result in serious complications after surgical interventions. Foreign materials such as implants or surgical sutures are optimal surfaces for the adherence of bacteria and subsequent colonization and biofilm formation. Due to a significant increase in antibiotic-resistant bacterial strains, naturally occurring agents exhibiting antibacterial properties have great potential in prophylactic therapies. The aim of this study was to develop a coating for surgical sutures consisting of the antibacterial substance totarol, a naturally occurring diterpenoid isolated from *Podocarpus* *totara *in combination with poly(lactide-*co*-glycolide acid) (PLGA) as a biodegradable drug delivery system. Hence, non-absorbable monofilament and multifilament sutures were coated with solutions containing different amounts and ratios of totarol and PLGA, resulting in a smooth, crystalline coating. Using an agar diffusion test (ADT), it became evident that the PLGA/totarol-coated sutures inhibited the growth of *Staphylococcus aureus *over a period of 15 days. A 3-(4,5-dimethylthiazol-2-yl)-2,5-diphenyltetrazolium bromide (MTT) assay showed that the coated sutures were not cytotoxic to murine fibroblasts. Overall, the data indicates that our innovative, biodegradable suture coating has the potential to reduce the risk of SSIs and postoperative biofilm-formation on suture material without adverse effects on tissue.

## 1. Introduction

Surgical site infections (SSIs) are one of the most critical parameters after surgical intervention, especially in contexts in which foreign materials such as implants or sutures are brought into the wound. Different bacteria are able to adhere and colonize on the surface of surgical sutures, causing infection. Surgical sutures are sterile stiches used to seal wounds after surgical procedures. They are an important tool to support wound healing [1,2], and the increased risk of SSIs compromises their usefulness. The most common pathogen causing these infections is *Staphylococcus aureus*, a gram-positive bacterium, which is responsible in 23% of the cases [3,4]. To reduce the incidence of SSIs, this source has to be eliminated [5]. One approach is to coat the surface of surgical sutures with antibacterial agents like antibiotics or other antibacterial substances in order to prevent bacterial adhesion on the surface without impairing other properties of the surgical suture. Such a coating can be implemented with the aid of poly(lactide-*co*-glycolide acid) (PLGA) in combination with an antibacterial active ingredient.

Biodegradable polymers, especially PLGA, have been used, mainly due to their good biodegradability, biocompatibility, and toxicological properties, as drug delivery systems in medicine and pharmacy. PLGA can be combined with a wide range of active ingredients, which are enclosed in PLGA and released over time during its degradation process [6,7,8].

Moreover, it is one of the few polymers that have been approved by the Food and Drug Administration (FDA) for clinical use in humans. Nowadays, PLGA is very widely used in systems with controlled drug release over a few days to months, including microspheres, nanoparticles, and implant coatings for local delivery [9,10].

In addition to antibiotics, natural antibacterial agents exist, which have great potential to be used as prophylactic agents against wound infections, mainly because antibiotic resistance may be circumvented. The active ingredient, totarol, a natural substance extracted from *Podocarpus totara*, demonstrates antibacterial activity against different bacteria, including *Staphylococcus aureus *(*S. aureus*) [11,12] and Methicillin-resistant *Staphylococcus aureus *(MRSA) [11,13]. So far, the exact mechanism of the antibacterial activity of totarol is not entirely clear. It is speculated that totarol inhibits bacterial respiratory transport [14], influences the multi drug efflux-pump [15], or disrupts the phospholipid membranes [16]. Another antibacterial impact is the targeting of the protein FtsZ to inhibit bacterial cytokinesis [17]. These properties are very interesting for future antibacterial therapies since totarol has low cytotoxicity [14,18,19].

Therefore, we hypothesize that, by combining PLGA and totarol into a biodegradable, antibacterial coating on sutures, a controlled release of the natural active substance and hence an inhibition of bacterial adhesion and biofilm formation on the material may be achieved. Our data indicate that totarol-coated sutures exhibit antibacterial activity against *S. aureus* in vitro over at least 15 days, while they do not induce adverse effects on the viability of fibroblasts.

## 2. Results

### 2.1. Antibacterial Activity of Totarol

#### Minimal Inhibitory Concentration (MIC)

**Figure 1:**
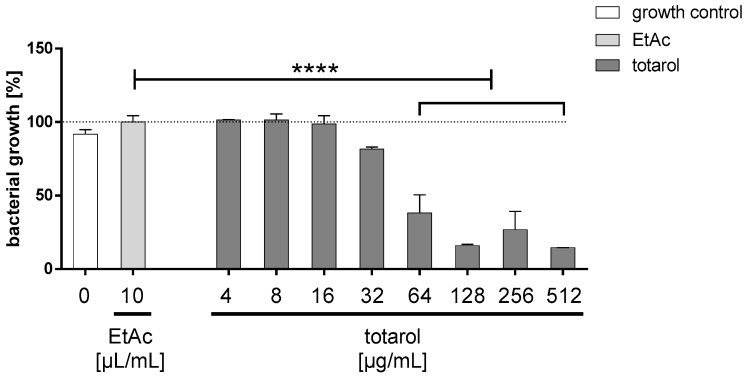
The Minimal Inhibitory Concentration (MIC) was established using the macrodilution method (*n = *3) according to the Clinical and Laboratory Standard Institute (CLSI) standard M07-A9. A significant decrease in bacterial growth after 24 h was detected when totarol concentrations of 64 µg/mL or higher were used. Data are given as means with standard error of the mean (SEM). One-way RM (repeated-measures) ANOVA followed by Tukey’s multiple comparisons test was used to identify significant differences between the varied totarol concentrations. **** indicates that the results are significantly different compared to the EtAc control (*p *< 0.0001). The white bar shows the growth control; the light grey bar depicts bacterial growth in the presents of EtAc and the dark grey bars display the growth of *S. aureus* exposed to different concentrations of totarol.

The antibacterial effect of purified totarol on *S. aureus* (ATCC 25923) was initially evaluated by MIC (Figure 1). Macroscopically, the turbidity of the inoculated totarol-laced media, indicating bacterial growth, became less pronounced at 64 µg/mL until the medium appeared clear at even higher concentrations of totarol. These findings were also verified by a photometric analysis, which indicates that the lowest concentration of totarol completely inhibiting the bacterial growth after 24 h was 128 µg/mL.

Additionally, there is no significant difference between the growth control and the ethyl acetate control, indicating that the solvent does not influence the growth of *S. aureus* (ATCC 25923).

### 2.2. Coating Analysis

**Figure 2:**
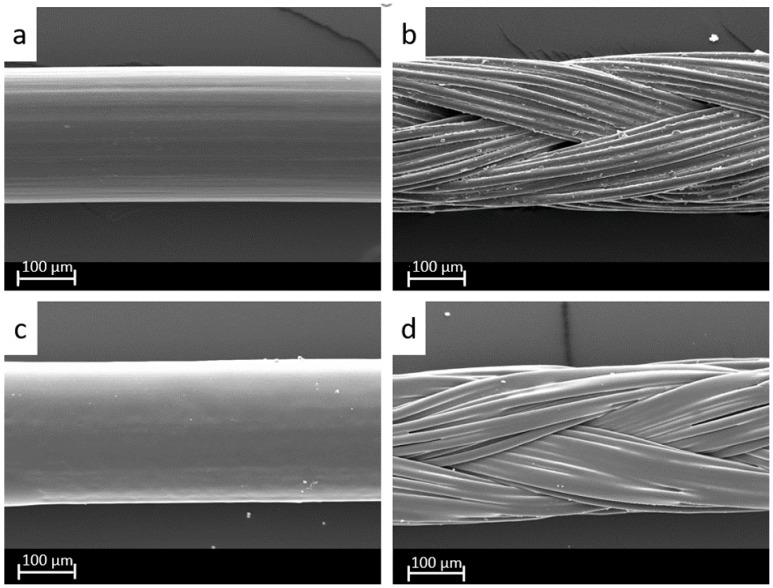
SEM investigation of (**a**) uncoated monofilament and (**b**) uncoated multifilament sutures as well as (**c**) totarol-poly(lactide-co-glycolide acid) (PLGA) (ratio 1:1) coated monofilament and (**d**) totarol-PLGA (ratio 1:1) coated multifilament sutures at 400× magnification.

The coating of the sutures was performed by spraying the uncoated monofilament (Figure 2a) as well as the multifilament (Figure 2b) sutures with the respective coating solution. The coating manifested as a relatively smooth layer, crystalline in appearance on all monofilament (Figure 2c) and multifilament (Figure 2d) sutures. With increasing concentrations of PLGA, the coating lost its crystalline characteristics. Sutures coated only with totarol had a coarser surface, white in color, which flaked with handling. To the touch, the threads were stiffer than the original suture and slightly sticky.

Table 1 depicts the various amounts of totarol and PLGA loaded on each suture and the composition of the corresponding stock solutions. Two different concentrations of totarol were used, 25 mg/mL and 100 mg/mL, in combination with different PLGA-concentrations of 0 mg/mL, 25 mg/mL, 50 mg/mL, and 75 mg/mL to prepare the stock solutions. After coating, varied amounts of totarol and PLGA were identified on the suture material after it dried. Despite applying coating solutions containing the same concentrations of totarol, i.e., 25 mg/mL or 100 mg/mL, the amounts coating the respective sutures differed slightly from each other. In addition, slight variations in the applied PLGA amounts were seen when comparing the sutures coated with stock solutions containing the same concentration of the polymer. The different quantities and ratios of both ingredients were used to later determine differences in the efficiency of the long-term application of antibacterial substances.

### 2.3. Long-Term Efficacy of Totarol

#### Agar Diffusion Test (ADT)

**Figure 3:**
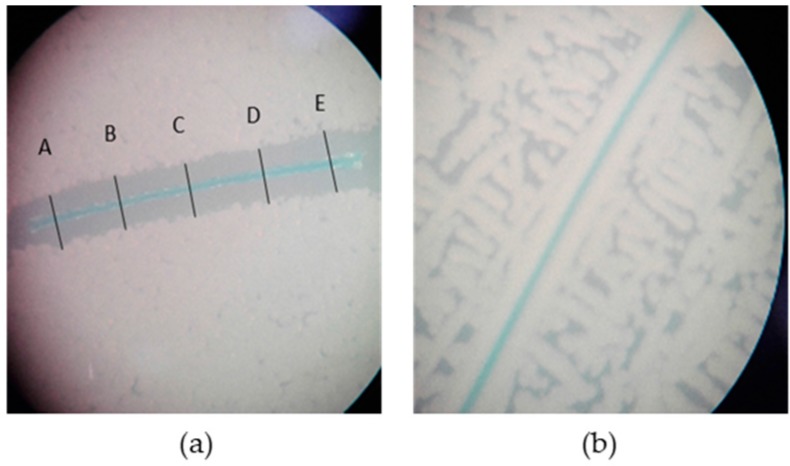
Multifilament sutures incubated with *S. aureus* at 37 °C for 24 h on Mueller-Hinton-agar: (**a**) depicts a totarol-PLGA coated suture with a clear zone of inhibition, measured at five different sites (A, B, C, D, E), and (**b**) shows an uncoated suture without a zone of inhibition. Mean diameter = [(A + B + C + D + E)/5] ± SEM.

The zones of inhibition were measured at five random sites (A–E) along the suture, as exemplarily shown in Figure 3a. No zone of inhibition was detectable when PLGA-coated or uncoated sutures were used in all experiments (Figure 3b).

**Figure 4:**
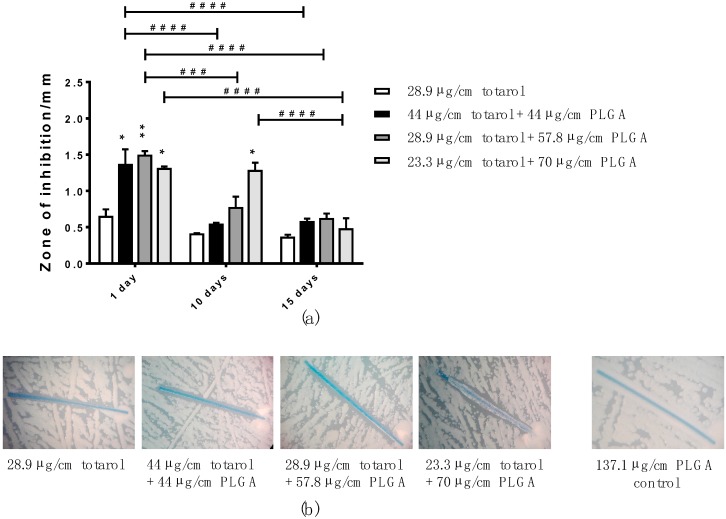

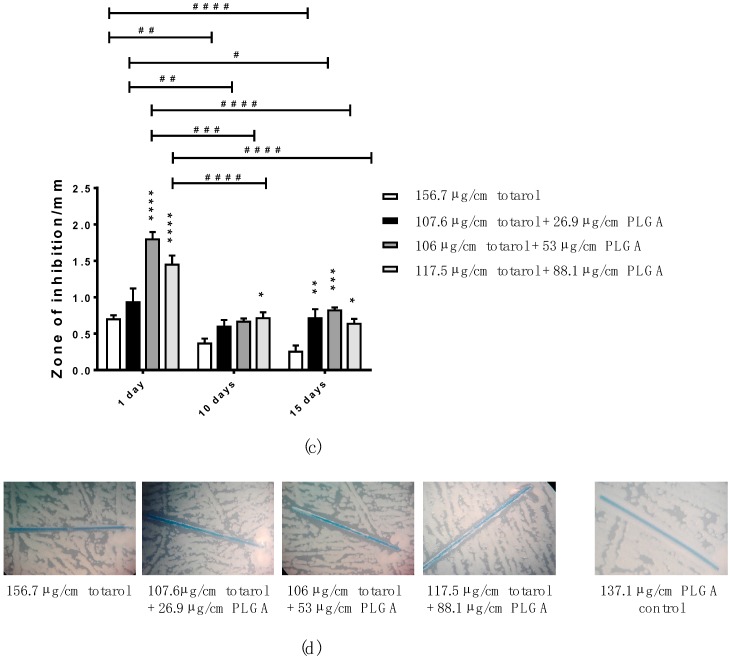
(**a**) Bar graph showing the differences in the zones of inhibition, arising during the Agar Diffusion Test (ADT), of monofilament sutures coated with a 25 mg/mL totarol stock solution and different concentrations of PLGA at days 1, 10 and 15 (*n = *3); (**b**) Representative images demonstrating a similar zone of inhibition after 15 days in all four groups. As the negative control, a PLGA-only-coated suture is shown; (**c**) Bar graph depicting the change in zones of inhibition around monofilament sutures sprayed with a coating solution with a concentration of 100 mg/mL totarol and varying amounts of PLGA at days 1, 10 and 15 of the ADT (*n = *3); (**d**) Representative images of the zones of inhibition of sutures coated in the four stock solutions at day 15. As the negative control, a PLGA-only-coated suture is shown. Data are given as means with SEM. Two-way RM ANOVA followed by Tukey’s multiple comparisons test was used to identify significant differences. Asterisks (* *p *< 0.05, ** *p *< 0.01, *** *p *< 0.001, and **** *p *< 0.0001) indicate statistical significances between the diverse coatings and the control (totarol coating without PLGA). Hashes (^#^ *p *< 0.05, ^##^* p *< 0.01, ^###^* p *< 0.001, and ^####^* p *< 0.0001) indicate statistical significance between the equal coating solutions at the various time points.

To determine the long-term efficacy of totarol-coated sutures on *Staphylococcus aureus*, an ADT was performed over a period of 15 days. The zones of inhibition induced by monofilament sutures coated with stock solutions containing 25 mg/mL totarol in combination with different amounts of PLGA are depicted in Figure 4a. The data indicates that the zones of inhibition at day 1 significantly increased when PLGA was used compared with sutures coated with totarol only. After 10 days of incubation, the sutures coated with 23.3 µg/cm totarol + 70 µg/cm PLGA were significantly different when compared to the other coatings. At day 15, the zones of inhibition were comparable in all four groups, which is also exemplarily shown in Figure 4b.

Figure 4c shows the performance of the monofilament suture material coated in stock solutions consisting of 100 mg/mL totarol and PLGA in varying amounts. Compared to the totarol-only-coated suture at day 1, the zones of inhibition are significantly bigger for sutures coated with 106 µg/cm totarol + 53 µg/cm PLGA and 117.5 µg/cm totarol + 88.1 µg/cm PLGA. After 10 and 15 days, the zones of inhibition on all sutures were significantly reduced. Coatings containing PLGA caused slightly larger zones of inhibition at 10 and 15 days than those containing only totarol. A significant difference was only seen in sutures coated with 117.5 µg/cm totarol + 88.1 µg/cm PLGA after 10 days and in all sutures containing any amount of PLGA after 15 days, as exemplarily shown in Figure 4d.

**Figure 5:**
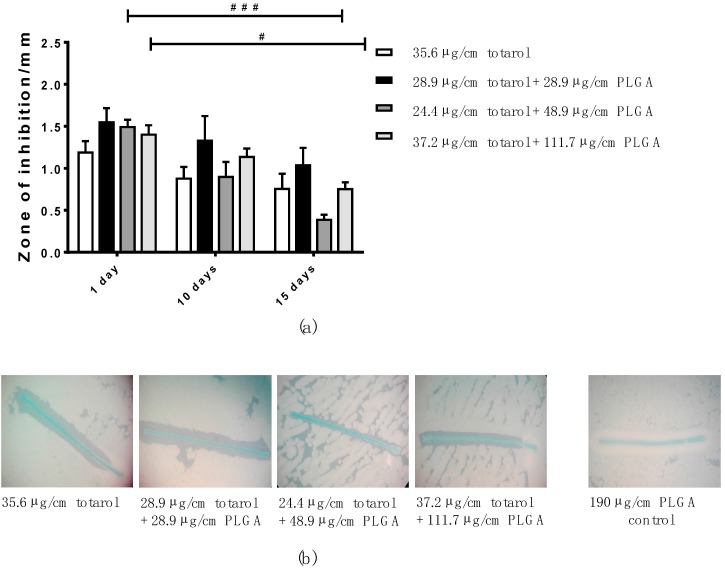

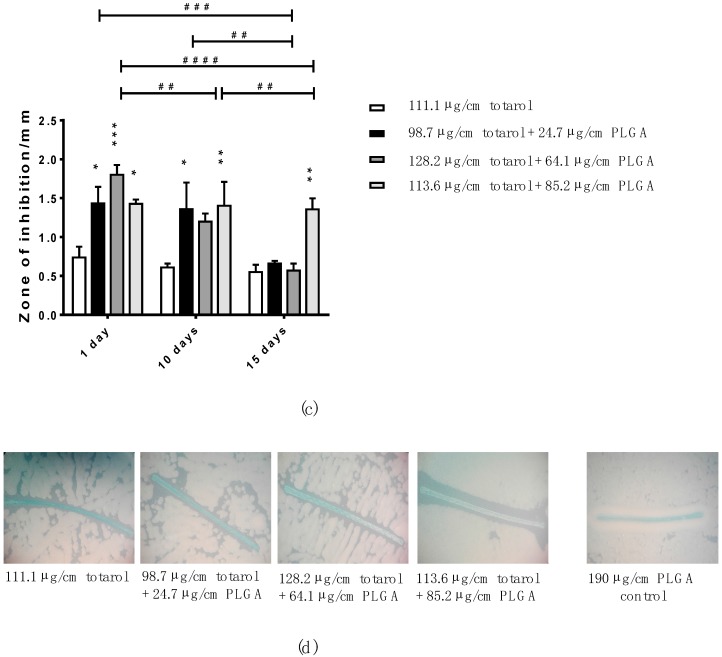
(**a**) Differences in the zones of inhibition of multifilament sutures coated in stock solutions made with 250 mg totarol and different amounts of PLGA at days 1, 10, and 15 of the ADT (*n = *3); (**b**) Representative images of the sutures coated in the four stock solutions and their zones of inhibition after 15 days. As the negative control, a PLGA-only-coated suture is shown; (**c**) Bar graph representing the characteristics of the zones of inhibition caused by multifilament suture material coated with a 100 mg/mL totarol stock solution combined with varying amounts of PLGA at days 1, 10, and 15 of the ADT (*n = *3); (**d**) Representative images of the different sutures and their zones of inhibition after 15 days. As the negative control, a PLGA-only-coated suture is shown. Data are given as means with SEM. Two-way RM ANOVA followed by Tukey’s multiple comparisons test was used to identify significant differences. Asterisks (* *p *< 0.05, ** *p * < 0.01, *** *p *< 0.001, and **** *p *< 0.0001) indicate statistical significances between the diverse coatings and the control (totarol coating without PLGA). Hashes (^#^ *p *< 0.05, ^##^ *p *< 0.01, ^###^ *p *< 0.001, and ^####^ *p *< 0.0001) indicate statistical significance between the equal coating solutions at the various time points.

In Figure 5a, the efficiency of multifilament sutures coated with a 25 mg/mL totarol stock solutions and different amounts of PLGA is shown. No significant difference in the diameters of the zones of inhibition of sutures additionally coated with PLGA and those coated only with totarol was detectable at any point during the experiment. Over time, the zones of inhibition mildly decreased. Although still visible, the zones of inhibition were significantly reduced for sutures coated with 24.4 µg/cm totarol + 48.9 µg/cm PLGA and 37.2 µg/cm totarol + 111.7 µg/cm PLGA compared to day 1. Figure 5b exemplarily depicts the zones of inhibition after 15 days.

Multifilament sutures containing PLGA displayed significantly larger zones of inhibition after one day than those only coated with the 100 mg/mL totarol solution (Figure 5c). After 10 days, the sutures coated with 98.7 µg/cm totarol + 24.7 µg/cm PLGA and 113.6 µg/cm totarol + 85.2 µg/cm PLGA still showed significantly larger zones of inhibition, while after 15 days only those coated with 113.6 µg/cm totarol + 85.2 µg/cm PLGA did. Here, the diameter was barely reduced during the experiment (Figure 5d). Again, during all experiments, uncoated sutures and sutures coated only with PLGA served as the controls. No zone of inhibition was detectable at any time point in the control sutures.

Overall, our data indicate that totarol-coated sutures effectively prevent the bacterial colonialization of the test material. After 15 days of investigation, the sutures coated with a stock solution of 100 mg/mL totarol and 75 mg/mL PLGA showed the greatest zones of inhibition.

### 2.4. Cytotoxicity of Coated and Uncoated Sutures

#### MTT (3-(4,5-Dimethylthiazol-2-yl)-2,5-Diphenyltetrazolium Bromide) Assay

**Figure 6:**
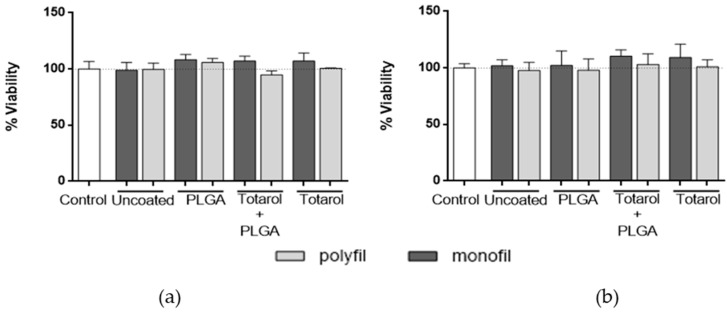
Cell viability of L929 fibroblasts exposed to differently coated sutures. The MTT (3-(4,5-Dimethylthiazol-2-yl)-2,5-Diphenyltetrazolium Bromide )-assay was performed (**a**) after 24 h at 37 °C without inhibiting cell growth and (**b**) after 48 h of incubation at 37 °C with no loss of viability. Data are given as means with SEM. Statistical analysis was done using the Friedman test. The measurements were normalized against the growth control and compared with it.

The cell viability assay was performed to examine whether totarol has cytotoxic effects on fibroblast strain L929 in the applied concentrations. The cells were subjected to the coated sutures over periods of 24 h and 48 h, respectively. As negative controls, uncoated and PLGA-only-coated suture materials were used. Compared to a growth control and uncoated monofilament and multifilament sutures, no reduction in the viability of the fibroblasts exposed to sutures coated with totarol (monofilament suture nr. 5: 156.7 µg/cm totarol, multifilament suture nr. 5: 111.1 µg/cm totarol, (Table 1)) or totarol in combination with PLGA (monofilament suture nr. 8: 117.5 µg/cm totarol and 88.1 µg/cm PLGA, multifilament suture nr. 8: 113.6 µg/cm totarol and 85.2 µg/cm PLGA (Table 1)) after 24 h (Figure 6a) and 48 h (Figure 6b) was detected. Furthermore, no adverse effects on morphology were visible.

## 3. Discussion

Surgical site infections are responsible for 15% of nosocomial infections and are associated with severe risks for patients [20]. Engelsmann et al. describe that 90% of these infections are caused by *S. aureus*, followed by *S. epidermidis*, *E. coli*, and *P. aeruginosa* [21]. Bacteria either enter the wound through incisions made during surgery or via contaminated foreign materials like surgical sutures. However, introducing these foreign materials into the surgical site exacerbates the risk of biofilm formation and subsequent SSIs [22], whereby a small amount of pathogens is sufficient to cause an infection compared to wounds without contact with foreign materials [23]. As a prophylactic measure against SSIs, caused by bacteria adhering to surgical sutures, antibacterial coatings are a promising strategy to reduce this hazard [22,24].

Nowadays, triclosan or silver are common coatings. However, exposure assessment shows that free triclosan, which is used in a wide variety of products like cosmetics, soaps, toys, kitchenware, and clothing is present in urine samples of 74.6% of the U.S. population and hence may have severe consequences on human health and contribute to bacterial resistance [25,26].

Therefore, the aim of this study was to implement a novel and safe biodegradable coating for surgical sutures, which is based on the naturally occurring antibacterial agent totarol. Totarol has previously been described as having antibacterial properties due to its varied mechanisms of action [11,12,14,18,27], which are not yet fully understood. It is highly probable that this versatility in function reduces the potential of bacteria to form resistance to the molecule, which is a serious problem with common antibiotics like triclosan [28]. The antibacterial effect of totarol works on several species of bacteria, among them *S. aureus* and MRSA (Methicillin-resistant *Staphylococcus aureus*) [12]. In this study, the negative effect on the growth of *S. aureus *was confirmed when performing the MIC assay. Opposed to previous studies [19], we used purified totarol and identified a MIC between 64 and 128 µg/mL, a lower concentration than that achieved with unpurified totarol [19] but higher than that described in earlier research [12]. Nevertheless, these results indicate that totarol may be a potent natural ingredient with which to coat surgical sutures and prevent the adherence of bacteria.

To be coated, non-absorbable monofilament and multifilament sutures were used and treated with different coating solutions with a varied ratio of totarol and PLGA. To examine the long-term antibacterial efficiency, the sutures were placed on a newly seeded agar plate every 48 h over a 15-day period. During the 15-day incubation period, macroscopic changes of the coating were visible, which might be attributed to the incubation temperature of 37 °C and the moisture in the petri dish. Exposure to water might cause swelling and the change of shape in PLGA over time [29]. Different drugs can also influence the behavior of the polymer, sometimes in a concentration dependent manner [29].

Overall, the measured zones of inhibition were small, which is mostly due to the poor water-soluble properties of totarol, resulting in poor diffusion of the agent. However, the results of the ADT clearly show that bacteria are not able to create a biofilm on the coated sutures when compared to uncoated or PLGA-coated sutures. Our data further indicate that the measured inhibition zones of the monofilament sutures are smaller than those of the multifilament sutures, which might be due to the different surface properties. The multifilament suture provides, due to its twisted design, a bigger surface for the coating. Moreover, the twisted structure ensures that the mixture of totarol and PLGA is absorbed more sufficiently via capillary forces and may also influence the drug release kinetic. Thereby, a constant drug release is possible with the multifilament structure, especially when compared to the monofilament structure, and may explain the greater zone of inhibition after 15 days.

Further examinations of sutures coated with silver nanoparticles were undertaken by Dhas et al. [30].

The impact of the coating was tested on *S. aureus *as well as *Pseudomonas aeruginosa*. While the antimicrobial efficacy was only determined for 24 h, the resulting zones of inhibition were similar in size to those observed with the Totarol-PLGA-coating deployed by us. In addition to silver, other molecules are also considered to coat sutures. Meghil et al. investigated a quaternary ammonium compound, K21, and its effect on *Enterococcus faecalis* and *Porphyromonas gingivalis* with positive results. The effects of the coated sutures on the bacteria strains were only tested until an even lawn of bacteria was grown [31]. Compared to the zones of inhibition achieved with Totarol-containing sutures, the resulting zones of inhibition were larger. In a previous study, the efficacy of octenidin-coated sutures compared to that of commercially available triclosan-coated sutures on *S. aureus* over a period of eight days was analyzed [32]. The antibacterial effect was comparable to that of Totarol in both instances. Obermeier et al. also examined the coated sutures for cytotoxicity. It was determined that the octenidin influenced the metabolic activity of L929 mouse fibroblasts negatively but within the acceptable parameters [32].

During our investigation, the cytotoxic effects of the totarol-coated sutures on human fibroblasts were excluded using the MTT assay. No change in viability or morphology could be observed. This is in accordance with a previous study in which no adverse effects of Totarol-loaded microspheres on fibroblasts were found [19]. Comparable results are also described for the cytotoxic effect of silver on fibroblasts [30].

## 4. Materials and Methods

### 4.1. Materials

PLGA (Resomer RG502H MW 7.000-17.000) was obtained from Evonik Industries AG (Essen, Germany). Ethyl acetate (EtAc, ACS grade), cerium-(IV)-sulfate, ammonium molybdate tetrahydrate, hexane, and Mueller Hinton broth were purchased from Sigma Aldrich (St. Louis, MO, USA). The active ingredient ‘totarol’ was from Santa Cruz (Dallas, TX, USA) and was also provided by aimecs® GmbH (Pfarrkirchen, Germany). Dimethyl sulfoxide (DMSO) was purchased from Merck KGaA (Darmstadt, Germany). MTT (3-(4,5-dimethylthiazol-2-yl)-2,5-diphenyltetrazolium bromide) was obtained from AppliChem (Darmstadt, Germany), and PBS (phosphate buffer saline), DMEM (Dulbecco’s Modified Eagle Medium), high glucose (with 10% fetal bovine serum, 4.5 mmol l-glutamine, 30 mmol HEPES (4-(2-hydroxyethyl)-1-piperazineethanesulfonic acid) and 5% penicillin-streptomycin), and RPMI (Roswell Park Memorial Institute) without phenol red media were from Gibco (Darmstadt, Germany). The monofilament suture material (Resonlon^®^, 75 cm USP 3/0) was obtained at Resorba^®^ (Nürnberg, Germany), and the multifilament sutures (Ethibond Excel, 75 cm, USP 3-0) were obtained from Ethicon (Johnson & Johnson Medical GmbH, Norderstedt, Germany). Petroleum ether was purchased from EMD Millipore (Billerica, MA, USA), and the silica gel (Silica 60 M, 0.04–0.063 mm) as well as the thin layer chromatography (TLC) plates were purchased from Macherey Nagel (Düren, Germany).

### 4.2. Totarol Purification

To determine the use of totarol as an antibacterial agent, the purchased substance first needed to be purified to remove possible cross-reactants. Purification was accomplished using column chromatography, as described previously [19]. Briefly, 7.5 g of totarol were dissolved in 8 mL of solvent. The solvent consisted of ethyl acetate and petroleum ether in a ratio of 1:20. The totarol sample was added to the column filled with the silica gel. Afterwards, 0.06 mbar of pressure was applied, and the run through solving agent was collected in fractions of 10 mL. Each fraction was tested on totarol purity using TLC by visualizing the sample and a control of pure totarol. Visualization occurred by applying air to the heat-sensitive molybdenum/cerium solution at 360 °C. Fractions holding pure totarol were added together, and the solvent was evaporated. Hexane was used to solve the crystallized totarol again, and the sample was cooled to −22 °C to recrystallize the agent. A final control was done using TLC.

### 4.3. Coating Process

Non-absorbable suture materials (multifilament and monofilament) were coated with several coating solutions containing different concentrations of totarol combined with different amounts of PLGA, which were dissolved in ethyl acetate (Table 1). The coating was carried out using an airbrush paint spray gun, followed by a fixation step using a heat tool at 100 °C. The amount of totarol on each suture was determined by weighing the dry coated sutures. The amount of totarol per cm suture was calculated by first computing the percentage of totarol in the coating solution and then determining the weight of totarol to which this percentage translates when applied to the complete weight of the coating. The amount of PLGA on a one centimeter piece of suture was determined by subtracting the weight of totarol on the suture from the weight of the coating.

All multifilament sutures were also pretreated by soaking them in ethyl acetate to remove the manufacturer’s coating.

The surgical sutures were examined using a scanning electron microscope (SEM; Zeiss LEO 1430, Oberkochern, Germany).

### 4.4. In Vitro Antibacterial Activity

#### 4.4.1. Minimal Inhibitory Concentration (MIC)

The MIC was performed using the macrodilution method with different concentrations ranging from 4 to 512 µg totarol per mL Mueller-Hinton-broth (MHB). The assay was carried out according to the standard M07-A9 recommended by the CLSI (Clinical and Laboratory Standard Institute). Therefore, an overnight culture of *Staphylococcus aureus* ATCC 25923 (DSMZ, Braunschweig, Germany) was adjusted to the McFarland standard 0.5 (BioMérieux, Marcy-l’Étoile, France), which corresponds to 1.5 × 10^8^ bacteria per mL. This suspension was diluted in a ratio of 1:150 with MHB and divided into several tubes, with 1 mL per tube. A concentration series of totarol dissolved in EtAc was prepared, and 20 µL of each concentration was added to one tube. Following that, the tubes were filled up with medium to 2 mL and incubated at 37 °C for 24 h. To measure the bacterial growth, a photometer at a wavelength of 625 nm was used. A 2 mL bacterial suspension containing 20 µL EtAc served as a control. The final bacterial concentration in each tube before incubation was 5 × 10^5^ bacteria. The lowest concentration of totarol, which inhibited the initial inoculum, is defined as the MIC.

#### 4.4.2. Agar Diffusion Test

The coated sutures were tested for their long-term antibacterial effect on bacteria with the agar diffusion test (ADT).

An overnight culture of *Staphylococcus aureus* ATCC 25923 was adjusted to the 0.5 McFarland standard. From this culture, 100 µL were seeded on Mueller Hinton agar plates. Subsequently, a 10 mm totarol-coated filament was placed on the plate, and the inhibition zone around the suture was investigated after 24 h of incubation at 37 °C. Uncoated suture material and sutures only coated with PLGA were used as negative controls. To examine their long-term efficiency, the sutures were applied to freshly seeded petri dishes every 48 h over a period of 15 days. The evaluation was performed by measuring the zone of inhibition every 24 h after each inoculation period. The zones of inhibition were measured at five random sites (A–E) along the suture, as exemplarily shown in Figure 3. The mean of the measurements at the five different locations represents the average diameter of the zone of inhibition.

### 4.5. Cytotoxicity of Coated and Uncoated Sutures

The coated and uncoated sutures were also tested for their cytotoxic properties. Therefore, an MTT assay was carried out according to the DIN EN ISO 10993-5 ‘Biological evaluation of medical devices—Part 5: Tests for in vitro cytotoxicity’ to determine cell viability after exposure to totarol and PLGA. L929 murine fibroblasts (DSMZ, Braunschweig, Germany) were seeded at a density of 75,000 cells per well in 12-well culture plates with DMEM medium. After incubation for 24 h at 37 °C and 5% CO_2_, the medium was removed and the cells were washed once with PBS before fresh medium was added. The totarol-coated sutures (monofilament suture nr. 5: 156.7 µg/cm totarol, multifilament suture nr. 5: 111.1 µg/cm totarol (Table 1)), the sutures containing totarol and PLGA (monofilament suture nr. 8: 117.5 µg/cm totarol and 88.1 µg/cm PLGA, multifilament suture nr. 8: 113.6 µg/cm totarol and 85.2 µg/cm PLGA (Table 1)), and the uncoated sutures were added to the corresponding wells and incubated for 24 h and 48 h, respectively. The medium and the sutures were removed, and the cells washed three times with PBS. Subsequently, 30 µL of a 0.5% MTT/PBS solution and 300 µL of RPMI medium were added to the individual wells and incubated for 4 h at 37 °C and 5% CO_2_. The supernatant was aspirated and replaced with 300 µL DMSO, followed by another incubation at 37 °C and 5% CO_2 _for 10 min. Afterwards, 10 µL were transferred to a 96-well plate already filled with 90 µL DMSO per well and measured at 540 nm with the BioTek EON^TM^ photometer (BioTek, Winooski, VT, USA). All experiments were carried out in triplicate.

### 4.6. Statistics

Data are depicted as means with SEM. The MIC data was analyzed using one-way repeated-measures (RM) ANOVA followed by Tukey’s multiple comparisons test. ADT data was analyzed using RM two-way ANOVA, with Tukey’s multiple comparison test to analyze the differences between groups. The MTT data was analyzed using the Friedman test to detect any potential significances between the groups. All analyses were performed using the statistical software package GraphPad Prism (version 6, GraphPad Software, La Jolla, CA, USA). Statistical significance was defined as *p *< 0.05.

## 5. Conclusions

This study analyzed the use and effectiveness of totarol as an antibacterial coating for suture material. Totarol-solutions and solutions containing both totarol and the polymer PLGA were used to successfully coat non-absorbable monofilament as well as multifilament sutures. It was shown that totarol has antibacterial properties as a purified substance in solution and that it retained those properties in vitro as a coating on its own and in combination with PLGA. The safest and most efficient combination of totarol and PLGA tested appeared to be the coating solution containing 100 mg/mL totarol and 75 mg/mL PLGA. The growth of *Staphylococcus aureus* around the totarol-coated material was inhibited in the long-term largely independently of its concentration and, therefore, can prevent biofilm formation in vitro. It was also demonstrated that totarol has no negative effect on the viability or morphology of murin fibroblasts in vitro.

In conclusion, our biodegradable totarol coating shows promise as a coating for sutures to prevent biofilm formation during the critical phase of wound healing and hence may decrease the occurrence of surgical site infections.

## Figures and Tables

**Table 1:** Amounts of totarol and PLGA in the coating solution and coated on the sutures.

Monofilament Sutures	m (Totarol) in Solution (mg/mL)	m (PLGA) in Solution (mg/mL)	Calculated Value Totarol Applied (µg/cm)	Calculated Value PLGA Applied (µg/cm)
**1**	25	---	28.9	---
**2**	25	25	44	44
**3**	25	50	28.9	57.8
**4**	25	75	23.3	70
**5**	100	---	156.7	---
**6**	100	25	107.6	26.9
**7**	100	50	106	53
**8**	100	75	117.5	88.1
**9**	---	75	---	137.1
**Multifilament Sutures**				
**1**	25	---	35.6	---
**2**	25	25	28.9	28.9
**3**	25	50	24.4	48.9
**4**	25	75	37.2	111.7
**5**	100	---	111.1	---
**6**	100	25	98.7	24.7
**7**	100	50	128.2	64.1
**8**	100	75	113.6	85.2
**9**	---	75	---	190

## Abbreviations

The following abbreviations are used in this manuscript:

SSI	Surgery site infection
PLGA	Poly(lactide- *co*-glycolide acid)
ADT	Agar diffusion test
MTT	3-(4,5-dimethylthiazol-2-yl)-2,5-diphenyltetrazolium bromide
FDA	Food and Drug Administration
*S. aureus*	*Staphylococcus aureus*
MRSA	Methicillin-resistant *Staphylococcus aureus*
MIC	Minimal inhibitory concentration
ATCC	American Type Culture Collection
CLSI	Clinical and Laboratory Standard Institute
EtAc	Ethyl acetate
DMSO	Dimethyl sulfoxide
PBS	Phosphate buffer saline
DMEM	Dulbecco’s Modified Eagle Medium
HEPES	4-(2-hydroxyethyl)-1-piperazineethanesulfonic acid
TLC	Thin layer chromatography
SEM	Scanning electron microscope
MHB	Mueller-Hinton-broth
CLSI	Clinical & Laboratory Standards Institute
SEM	error of the mean
RM	repeated-measures
